# A Novel Prognostic Nomogram Based on TIGIT and NKG2A Can Predict Relapse‐Free Survival of Hepatocellular Carcinoma After Hepatectomy

**DOI:** 10.1002/cam4.70419

**Published:** 2024-11-14

**Authors:** Junqi Wang, Yuqing Cao, Yu Tian, Chaoliu Dai, Tianqiang Jin, Feng Xu

**Affiliations:** ^1^ Department of General Surgery Shengjing Hospital of China Medical University Shenyang Liaoning China; ^2^ Department of Critical Care Medicine The Fifth People's Hospital of Zhangjiagang City Suzhou Jiangsu China

**Keywords:** hepatocellular carcinoma, NKG2A, prognosis, prognostic model, TIGIT

## Abstract

**Background and Objectives:**

Hepatocellular carcinoma (HCC) is a major global health concern, and emerging evidence suggests that TIGIT and NKG2A are potential immune checkpoints with implications for HCC progression. This study aimed to evaluate the prognostic significance of TIGIT and NKG2A expression in HCC patients who underwent radical liver resection.

**Methods:**

We conducted a retrospective analysis of 144 HCC patients who underwent radical liver resection. TIGIT and NKG2A expression levels were assessed using the immunoreactive score. Cox proportional hazards models were utilized to analyze the association between TIGIT/NKG2A expression and clinical characteristics, relapse‐free survival (RFS), and overall survival (OS). Prognostic models for OS and RFS was developed and validated using concordance index and calibration curves. Additionally, the random forest algorithm was employed to identify independent risk factors for OS and RFS and their correlation with predicted survival.

**Results:**

TIGIT and NKG2A expression were identified as independent risk factors for RFS, while TIGIT expression alone significantly impacted OS. The prognostic models showed good discriminative ability, with concordance indices exceeding 0.7 for predicting 1‐, 3‐, and 5‐year OS or RFS. Calibration curves confirmed the reliability of the nomograms for OS and RFS prediction. The areas under the ROC curve consistently exceeded 0.7 for predicting OS and RFS. Elevated levels of TIGIT and NKG2A expression were associated with diminished RFS, highlighting their importance as prognostic factors.

**Conclusions:**

Our study establishes the prognostic significance of TIGIT and NKG2A expression in predicting OS and RFS following radical liver resection for HCC patients. The developed prognostic models incorporating TIGIT and NKG2A expression hold promise for improving risk stratification and clinical management of HCC patients.

## 1 Introduction

Hepatocellular carcinoma (HCC) represents a prevalent malignancy globally, ranking as the third leading cause of cancer‐related motality [1]. Despite emerging consensus on standard treatment protocols for HCC, including surgical resection, ablation, transplantation, locoregional, and systemic treatments, the prognosis for HCC patients remains unsatisfactory [2, 3, 4].

The intricate interplay between protective immunity and immune tolerance within the liver contributes to the observed poor prognosis in HCC [5]. Immune dysfunction, a hallmark feature of HCC, enables tumor cells to evade immune surveillance through various mechanisms [6, 7]. Among these, the interaction between immune checkpoint ligands on tumor cells and receptors on immune cells plays a pivotal role in facilitating immune escape [8]. In recent years, immune checkpoint modulation has emerged as a promising therapeutic avenue in cancer research. Key immune checkpoint molecules including programmed cell death protein 1 (PD‐1), programmed cell death 1 ligand 1 (PD‐L1), cytotoxic T‐lymphocyte‐associated protein 4 (CTLA‐4), T cell immunoglobulin and mucin domain 3 (TIM‐3) and lymphocyte‐activation gene 3 (LAG‐3) have garnered significant attention [9]. Although monoclonal antibody therapy targeting these molecules has demonstrated promise in improving HCC prognosis, its widespread application remains limited [10].

Recently, two novel immune checkpoint molecules, T cell immunoreceptor with Ig and immunoreceptor tyrosine‐based inhibition motif (ITIM)domains (TIGIT), and natural‐killer group 2 member A (NKG2A), have emerged as potential prognostic markers in certain cancers [11]. In this study, we aimed to assess the prognostic significance of TIGIT and NKG2A expression in HCC patients undergoing radical resection. Additionally, we sought to establish a prediction model based on independent risk factors identified from our data to explore its utility in predicting the prognosis of HCC patients.

## 2 Material and Methods

### 2.1 Dataset

We conducted a retrospective analysis of a dataset comprising all patients diagnosed with HCC who underwent liver resection at the Department of General Surgery, Shengjing Hospital of China Medical University, between March 2014 and December 2019. Inclusion criteria consisted of first‐onset cases, complete resection of liver tumors, histopathological confirmation of HCC, absence of preoperative extrahepatic metastasis, and availability of complete clinical and follow‐up data. The following criteria were used for exclusion: (a) receipt of other preoperative treatments, (b) immune disease, (c) combined with ablation, (d) presence of other tumor, (e) abnormal renal or cardiovascular function. Patients meeting the inclusion and exclusion criteria were enrolled in our study.

### 2.2 Immunohistochemistry

The formalin‐fixed paraffin‐embedded HCC tissue blocks were sectioned into 4‐μm slices and mounted onto glass slides. The slides underwent deparaffinized in xylene, followed by rehydration in a graded series of ethanol and washing with distilled water. Subsequently, the tissue sections were immersed in phosphate‐buffered saline (PBS). Heat‐induced epitope retrieval was performed under high temperature and pressure, followed by treatment with a peroxidase blocker to inhibit endogenous peroxidase activity. After incubation with an appropriate amount of primary antibody at 37°C for 60 min, the sections were washed three times with PBS for 3 min each. Next, the sections were incubated with an appropriate amount of enzyme‐labeled goat anti‐mouse/rabbit IgG polymer at room temperature for 20 min, followed by another three washes with PBS for 3 min each. The freshly prepared color‐developing solution was applied to the sections, followed by a 5‐min incubation period at room temperature. Finally, the sections were counterstained with hematoxylin, dehydrated in ethanol, cleared in xylene, and covered with cover slips. The mouse anti‐human TIGIT monoclonal antibody was purchased from Dianova (Hamburg, Germany), while the rabbit anti‐human NKG2A monoclonal antibody and second antibody were purchased from Abcam (Cambridge, UK). Other immunohistochemical reagents were sourced from Fuzhou Maixin Biotech (Fujian, China).

### 2.3 Immunohistochemical Evaluation

The immunohistochemical sections of all tumor specimens were independently double‐blindly observed by two senior pathologists at our hospital. The expression of TIGIT and NKG2A is manifested as yellow to tan particles on the cell membrane. To assess protein staining in HCC tissue, we employed an immunoreactive score (IRS), calculated by multiplying the staining intensity with the percentage of positively stained cells [12, 13]. The specific details of the IRS are as follows: the staining intensity was categorized into four gradations: absent (grade 0), light yellow (grade 1), brown (grade 2), and tan (grade 3). Positively stained cells were graded based on the percentage of cells: 0 (grade 0), ≤ 10% (grade 1), 11%–50% (grade 2), 51%–80% (grade 3), and > 80% (grade 4). The product of these two grades ranged from 0 to 12 points. Samples with an IRS ≤ 4 were classified as exhibiting low expression, while those with IRS > 4 were classified as exhibiting high expression. Five fields at 200× magnification were randomly selected for each sample.

### 2.4 Follow‐Up

All patients underwent follow‐up either by telephone or outpatient clinic. The initiation of follow‐up was marked by the enrollment date, while the conclusion of the follow‐up was represented by the cut‐off date set at October 31, 2020, or until the occurrence of death or recurrence. The study endpoints included overall survival (OS) and relapse‐free survival (RFS). OS was defined as the duration from the diagnosis of HCC to either patient death or the end of follow‐up, while RFS was defined as the period from the diagnosis of HCC to the detection of recurrence or the end of follow‐up.

### 2.5 Statistical Analysis

All data were analyzed using SPSS version 26.0.0.0 software, with statistical significance set at *p* < 0.05. Associations between categorical variables were evaluated using the Chi‐square test, Fisher's exact test, or Wilcoxon rank‐sum test, as appropriate. Univariate and multivariate analyses were conducted using the Cox proportional hazard model. The R Project version 4.1.3 was utilized to generate all figures, calculate the concordance index, and determine the areas under the ROC curve.

## 3 Results

### 3.1 Patient Clinical Characteristics

The study included a total of 144 patients diagnosed with HCC between March 2014 and December 2019, all of whom underwent radical resection. The median age of the patients was 58 years, ranging from 28 to 78 years old, with 103 (71.5%) being male. A total of 121 cases (84.0%) were diagnosed with a single tumor. Among these, 97 (76.4%) had tumors with a maximum diameter ≤ 5 cm, while 47 (23.6%) had tumors larger than 5 cm. The majority of tumors exhibited moderate to well differentiation (86.8%), were classified as BCLC stage A (72.2%), and had a Child‐Pugh grade A (92.4%). Further details regarding the clinical characteristics of patients were provided in Table 1.

**TABLE 1:** patient baseline characteristics.

Variables	*n* (%)
Age (< 60/≥ 60 years)	82 (56.9%)/62 (43.1%)
Gender (male/female)	103 (71.5%)/41 (28.5%)
BMI (< 24/≥ 24)	77 (53.5%)/67 (46.5%)
Tumor number (single/multiple)	121 (84.0%)/23 (16.0%)
Tumor size (≤ 5/> 5 cm)	97 (67.4%)/47 (32.6%)
Vascular cancer embolus (absent/present)	132 (91.7%)/12 (8.3%)
Differentiation (poor/moderate‐poor/moderate/high‐medium/well)	6 (4.2%)/13 (9.0%)/83 (57.6%)/13 (9.0%)/29 (20.1%)
Intraoperative blood loss (< 400/≥ 400 mL)	104 (72.2%)/40 (27.8%)
ASA grade (I/II/III)	12 (8.3%)/108 (75.0%)/24 (16.7%)
BCLC stage (0/A/B/C)	15 (10.4%)/104 (72.2%)/13 (9.0%)/12 (8.3%)
Child‐Pugh grade (A/B)	133 (92.4%)/11 (7.6%)
Liver cirrhosis (absent/present)	14 (9.7%)/130 (90.3%)
Types of hepatitis (absent/HBV/HCV/HBV + HCV/steatohepatitis)	8 (5.6%)/121 (84.0%)/5 (3.5%)/7 (4.9%)/3(2.1%)
Hypersplenism (absent/present)	97 (67.4%)/47 (32.6%)
AFP level (< 400/≥ 400 ng/mL)	104 (72.2%)/40 (27.8%)
Absolute value of lymphocyte (< 1.74/≥ 1.74 × 10^9^/L)	92 (63.9%)/52 (36.1%)

### 3.2 Analysis of TIGIT and NKG2A Expression and Correlation

**FIGURE 1:**
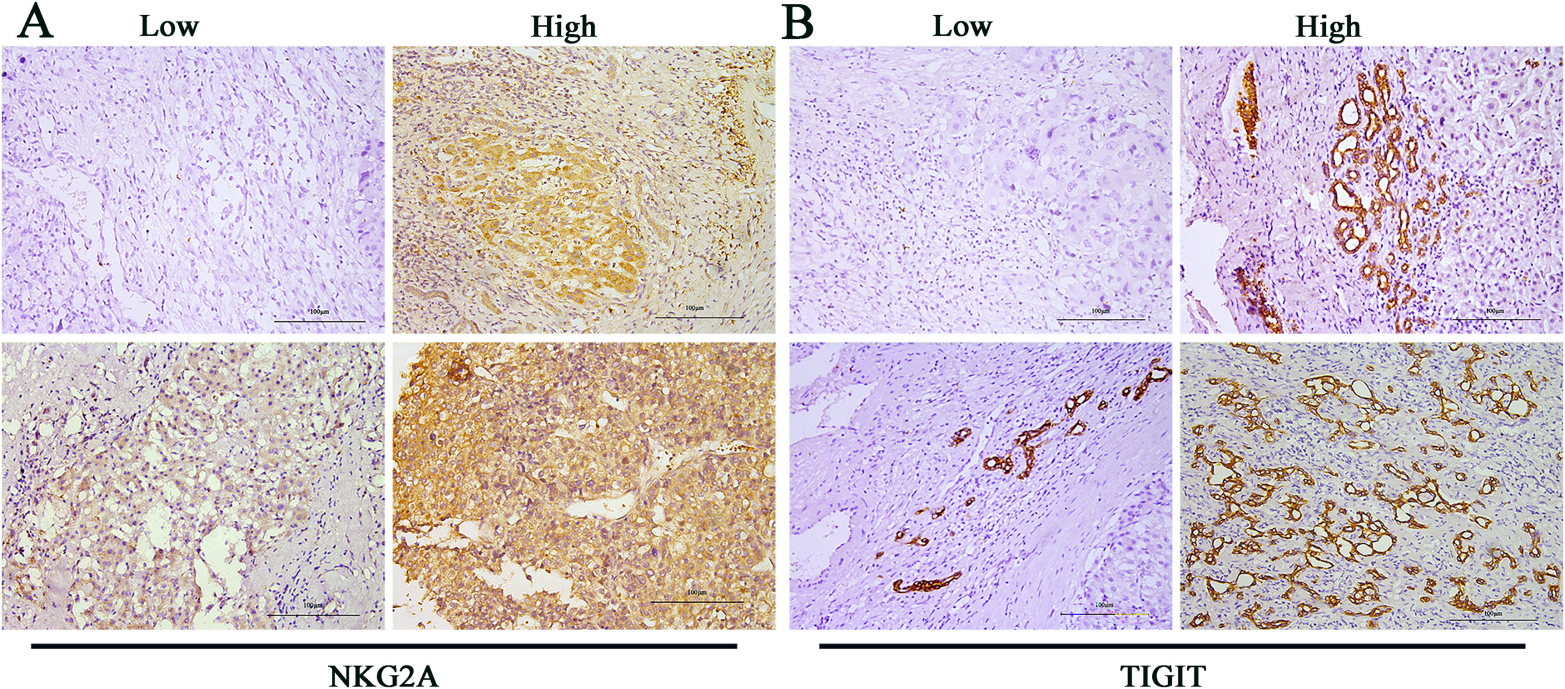
NKG2A and TIGIT expression in HCC tissue samples. (A) Representative cases of NKG2A expression within the tumor. (B) Representative cases of TIGIT expression within the tumor. All images are in a 200× magnification.

TIGIT protein expression was observed on the cell membrane and assessed using the aforementioned IRS score, as was NKG2A. Representative photomicrographs of TIGIT and NKG2A‐stained biospecimens were presented in Figure 1. According to our criteria, we observed that 66 cases (45.8%) showed low TIGIT expression (TIGIT^−^), while 78 cases (54.2%) exhibited high TIGIT expression (TIGIT^+^). Similarly, 77 cases (53.5%) demonstrated low NKG2A expression (NKG2A^−^) while 67 cases (46.5%) exhibited high NKG2A expression (NKG2A^+^). Further analysis revealed no significant correlation between TIGIT and NKG2A expression among the recruited patients (*p* = 0.242, Table S1). Therefore, our findings indicate that the expression of TIGIT and NKG2A does not affect each other, suggesting that they can serve as independent indicators for subsequent studies.

### 3.3 Association of Expression Level of TIGIT and NKG2A With Clinical Characteristics in HCC

Using the Chi‐square test, we investigated whether expression levels of TIGIT and NKG2A were associated with various clinical features in HCC. The Wilcoxon test was employed for ASA grade, BCLC stage, and Child‐Pugh grade, while the Fisher test was utilized for hepatitis types. Our analysis revealed no significant correlation between the expression levels of TIGIT and NKG2A and any of the clinical features examined, including gender, age, and BMI (*p* > 0.05). This suggests that there is no interaction between the observed clinical characteristics and the expression of TIGIT and NKG2A, hereby affirming their utility as independent factors for the prognosis analysis of patients. Detailed data were provided in Table S2.

### 3.4 Impact of TIGIT and NKG2A Expression on RFS and OS in HCC

**FIGURE 2:**
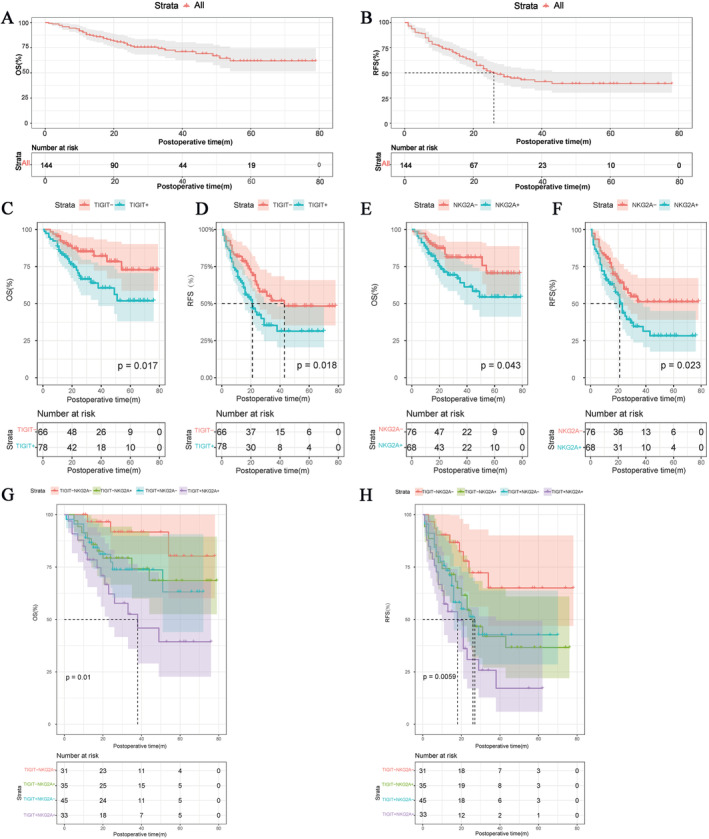
Kaplan Meier analysis of OS and RFS. (A) Kaplan Meier analysis for OS of the entire cohort. (B) Kaplan Meier analysis for RFS of the entire cohort. (C) Postoperative OS Kaplan–Meier curves based on TIGIT expression. (D) Postoperative OS Kaplan–Meier curves based on NKG2A expression. (E) Postoperative RFS Kaplan–Meier curves based on TIGIT expression. (F) Postoperative RFS Kaplan–Meier curves based on NKG2A expression. (G) Postoperative OS Kaplan–Meier curves based on TIGIT and NKG2A expression. (H) Postoperative RFS Kaplan–Meier curves based on TIGIT and NKG2A expression.

Given the limited significance of TIGIT and NKG2A expression for clinical characteristics, we proceeded to investigate their impact on the RFS and OS in HCC. Statistical analysis revealed that among the 144 recruited patients, 31 had died and 71 had experienced relapse by the end of the follow‐up period. The median RFS was determined to be 26.0 months. As the median OS was not reached, we were only able to ascertain that the average postoperative OS was 58.5 ± 2.8 months (Figure 2A,B).

Compared to the TIGIT^+^ group, the TIGIT^−^ group exhibited prolonged postoperative average RFS and OS (OS: 65.3 ± 3.5 vs. 50.7 ± 3.9 months, *p* = 0.017, HR: 0.45, 95% CI: 0.24–0.85; RFS: 47.1 ± 4.4 vs. 31.9 ± 3.6 months, *p* = 0.018, HR: 0.57, 95% CI: 0.36–0.9; Figure 2C,D). Similarly, both the postoperative average RFS and OS of the NKG2A^−^ group were longer than those of the NKG2A^+^ group (OS: 63.5 ± 3.5 vs. 53.1 ± 4.1 months *p* = 0.043, HR: 0.51, 95% CI: 0.27–0.97; RFS: 47.5 ± 4.2 vs. 32.8 ± 3.9 months, *p* = 0.023, HR: 0.59, 95% CI: 0.37–0.94; Figure 2E,F).

Considering the positive correlation between TIGIT/NKG2A expression with RFS and OS, we further categorized patients into four groups (TIGIT^−^NKG2A^−^/TIGIT^−^NKG2A^+^/TIGIT^+^NKG2A^−^/TIGIT^+^NKG2A^+^) based on both TIGIT and NKG2A expression to explore the impact of this co‐expression on RFS and OS. Our data revealed that postoperative average OS followed the sequence of TIGIT^−^NKG2A^−^>TIGIT^−^NKG2A^+^>TIGIT^+^NKG2A^−^>TIGIT^+^NKGA2^+^ (OS: 70.4 ± 4.1 vs. 61.0 ± 5.1 vs. 54.7 ± 4.5 vs. 43.3 ± 5.8, *p* = 0.010; Figure 2G), as did RFS (RFS: 57.3 ± 6.1 vs. 38.7 ± 5.5 vs. 37.7 ± 4.8 vs. 23.1 ± 4.1 months, *p* = 0.0059; Figure 2H). As expected, high expression levels of both TIGIT and NKGA2 were associated with a negative impact on HCC patients after radical resection, suggesting a certain synergistic effect between the two.

### 3.5 Univariate and Multivariate Analysis for HCC Prognosis

To identify independent risk factors influencing the prognosis of patients with HCC after surgery, we employed the Cox proportional hazards regression model for both univariate and multivariate analyses. In the univariate analysis, several factors emerged as significant negative predictive factors for postoperative RFS, including TIGIT^+^(*p* = 0.021), NKG2A^+^(*p* = 0.026), intraoperative blood loss ≥ 400 mL (*p* = 0.003), tumor size ≥ 5 cm (*p* = 0.014), presence of vascular tumor embolus (*p* < 0.001), absolute value of lymphocyte count (*p* = 0.024), and BCLC stage B‐C (*p* < 0.001). Subsequent multivariate analysis revealed that independent risk factors for postoperative RFS comprised TIGIT expression (*p* = 0.006), NKG2A expression (*p* = 0.042), tumor differentiation degree (*p* < 0.05), AFP level (*p* = 0.042), absolute value of lymphocyte count (*p* = 0.012), and BCLC stage (*p* = 0.023).

As shown in Table 2, multivariate analysis demonstrated that only TIGIT expression (*p* = 0.047), tumor size (*p* = 0.018), and AFP level (*p* = 0.015) were identified as independent risk factors influencing postoperative OS. Regarding postoperative RFS, univariate analysis revealed significant differences among patients with varying TIGIT expression (*p* = 0.021), NKG2A expression (*p* = 0.047), intraoperative blood loss (*p* = 0.011), tumor size (*p* < 0.001), presence of vascular tumor embolus (*p* = 0.002), AFP level (*p* < 0.001), and BCLC stage (*p* = 0.004). Detail data are provided in Table 3.

**TABLE 2:** COX analysis of related factors of postoperative OS.

Factors	Mean OS (m)	Univariate			Multivariate		
		HR	95% CI	*p*	HR	95% CI	*p*
TIGIT^−^/TIGIT^+^	65.282/50.748	1.000/2.247	1.132–4.461	**0.021** ^*^	1.000/2.047	1.011–4.146	**0.047** ^*^
NKG2A^−^/NKG2A^+^	63.466/53.147	1.000/1.948	1.008–3.768	**0.047** ^*^	1.000/1.846	0.944–3.610	0.073
Gender (female/male)	50.404/61.039	1.000/0.654	0.334–1.281	0.216			
Age (< 60/≥ 60years)	62.783/49.262	1.000/1.765	0.928–3.354	0.083			
BMI (< 24/≥ 24)	54.398/62.731	1.000/0.628	0.325–1.216	0.168			
ASA grade							
I	74.714	1.000					
II	57.059	4.161	0.563–30.766	0.162			
III	45.432	7.596	0.967–59.646	0.054			
Intraoperative blood loss							
(< 400/≥ 400 mL)	63.004 /47.211	1.000/2.290	1.210–4.332	**0.011** ^*^	1.000/1.472	0.715–3.031	0.294
Tumor number							
(single/ multiple)	58.103/48.826	1.000/0.947	0.369–2.432	0.910			
Tumor size (≤ 5/> 5 cm)	65.488 /41.693	1.000/3.401	1.783–6.489	< **0.001** ^*^	1.000/2.508	1.174–5.356	**0.018** ^*^
Vascular tumor embolus							
(absent/present)	60.423/30.833	1.000/3.653	1.605–8.314	**0.002** ^*^	1.000/2.270	0.610–8.452	0.222
Differentiation							
Poor	42.333	1.000					
Poor‐moderate	54.500	0.471	0.105–2.105	0.324			
Moderate	57.102	0.496	0.149–1.654	0.254			
Moderate‐high	48.275	0.400	0.081–1.984	0.262			
Well	63.048	0.292	0.070–1.225	0.093			
Hypersplenism							
(absent/present)	60.564/46.737	1.000/1.351	0.697–2.619	0.373			
AFP level							
(< 400/≥ 400 ng/mL)	64.869/41.302	1.000/3.386	1.789–6.406	< **0.001** ^*^	1.000/2.301	1.176–4.501	**0.015** ^*^
Absolute value of lymphocyte							
(< 1.74/≥ 1.7 × 10^9^/L)	53.120 /64.965	1.000/0.479	0.232–0.993	**0.048** ^*^	1.000/0.625	0.294–1.328	0.221
Child‐Pugh grade (A/B)	59.756 /41.244	1.000/2.565	0.996–6.606	0.051			
BCLC stage (0‐A/B‐C)	61.256/38.252	1.000/2.847	1.405–5.767	0.004*	1.000/1.587	0.539–4.674	0.402

*Note:* Statistically significant of *P* < 0.05.

**TABLE 3:** COX analysis of related factors of postoperative RFS.

Factors	Mean RFS (m)	Univariate			Multivariate		
		HR	95% CI	*p*	HR	95% CI	*p*
TIGIT^−^/TIGIT^+^	47.074 /31.917	1.000/1.765	1.090–2.857	**0.021** ^*^	1.000/2.032	1.220–3.384	**0.006** ^*^
NKG2A^−^/NKG2A^+^	47.521/32.807	1.000/1.708	1.066–2.735	**0.026** ^*^	1.000/1.667	1.018–2.730	**0.042** ^*^
Gender (female/male)	37.389/39.969	1.000/1.047	0.613–1.789	0.866			
Age (< 60/≥ 60years)	41.807/36.087	1.000/1.015	0.634–1.623	0.952			
BMI (< 24/≥ 24)	37.174/43.208	1.000/0.727	0.452–1.167	0.186			
ASA grade							
I	54.263	1.000					
II	37.803	1.912	0.690–5.296	0.216			
III	32.657	2.070	0.674–6.358	0.203			
Intraoperative blood loss							
(< 400/≥ 400 mL)	46.492/26.731	1.000/2.047	1.274–3.287	**0.003** ^*^	1.000/1.667	0.981–2.833	0.059
Tumor number							
(single/ multiple)	41.336/25.391	1.000/1.430	0.781–2.618	0.247			
Tumor size (≤ 5/> 5 cm)	45.726 /26.939	1.000/1.821	1.132–2.932	**0.014** ^*^	1.000/1.011	0.568–1.797	0.971
Vascular tumor embolus							
(absent/present)	42.655/9.250	1.000/4.319	2.187–8.529	**< 0.001** ^*^	1.000/2.494	0.941–6.610	0.066
Differentiation							
Poor	16.667	1.000			1.000		
Poor‐moderate	44.513	0.250	0.076–0.825	**0.023** ^*^	0.193	0.051–0.722	**0.015** ^*^
Moderate	42.379	0.306	0.120–0.783	**0.013** ^*^	0.260	0.090–0.750	**0.013** ^*^
Moderate‐high	25.750	0.402	0.131–1.235	0.111	0.412	0.118–1.433	0.163
Well	39.318	0.312	0.112–0.873	**0.026** ^*^	0.291	0.090–0.940	**0.039** ^*^
Types of hepatitis							
Absent	21.500	1.000					
HBV	40.395	0.798	0.320–1.989	0.628			
HCV	59.250	0.279	0.033–2.389	0.244			
HBV + HCV	30.810	0.771	0.184–3.231	0.722			
Steatohepatitis	33.000	0.771	0.148–4.008	0.757			
Hypersplenism							
(absent/present)	40.704/32.913	1.000/0.852	0.511–1.420	0.539			
AFP level							
(< 400/≥ 400 ng/mL)	44.228/30.078	1.000/1.639	1.001–2.682	**0.049** ^*^	1.000/1.790	1.023–3.135	**0.042** ^*^
Absolute value of lymphocyte							
(< 1.74/≥ 1.7 × 10^9^/L)	32.955/49.292	1.000/0.553	0.330–0.926	0.024*	1.000/0.496	0.286–0.859	0.012*
Child‐Pugh grade (A/B)	41.523 /16.788	1.000/1.786	0.814–3.918	0.148			
BCLC stage (0‐A/B‐C)	44.596/14.887	1.000/3.485	2.035–5.969	< **0.001** ^*^	1.000/2.477	1.132–5.421	0.023*

*Note:* Statistically significant of *P* < 0.05.

### 3.6 Establishment of a Prediction Model for RFS and OS

**FIGURE 3:**
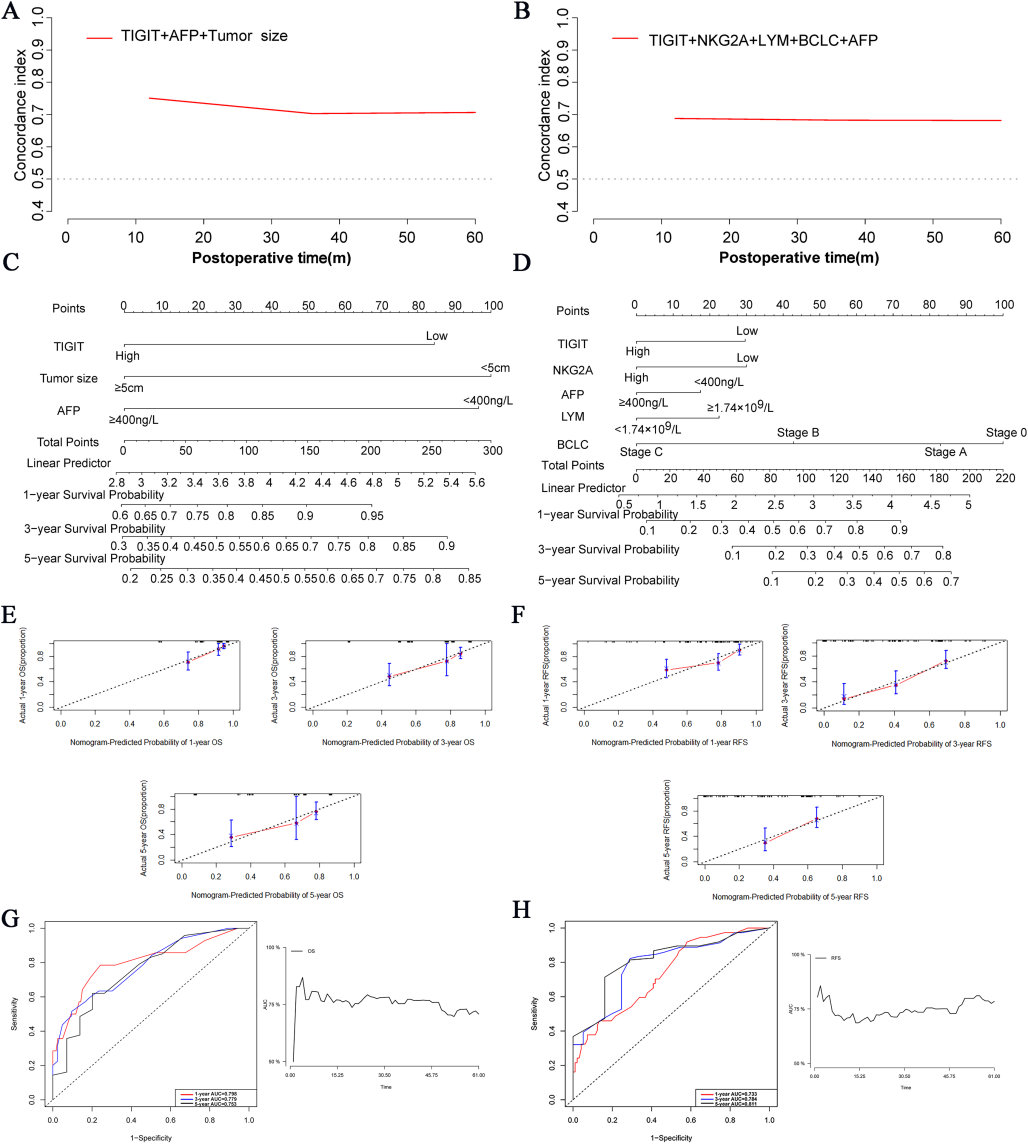
Establishment of prognostic models for OS and RFS. (A) Concordance index based on OS independent risk factors. (B) Concordance index based on RFS independent risk factors. (C) Nomogram for predicting 1‐,3‐,5‐year OS. (D) Nomogram for predicting 1‐, 3‐, 5‐year RFS. (E) Calibration curve for OS Nomogram. (F) Calibration curve for RFS Nomogram. (G) ROC curves predicting 1‐,3‐ and 5‐year OS and internal validation. (H) ROC curves predicting 1‐,3‐ and 5‐year RFS and internal validation.

Having identified the independent risk factors for both RFS and OS, our next step was to utilize these factors to establish a prognostic model to predict patient outcomes. The independent factors (TIGIT expression, AFP level, and tumor size) determined by multivariate analysis to impact postoperative OS were incorporated into the calculation of the concordance index. At the time intervals of 1, 3, and 5 years, the concordance index of the prediction model was 0.751, 0.703, and 0.706, respectively. Using the same method, we also developed a prediction model for RFS and presented all concordance indices graphically (Figure 3A,B). Subsequently, we integrated the derived prognostic models into a nomogram for predicting 1‐, 3‐, and 5‐year OS and RFS (Figure 3C,D). To assess the reliability of the nomogram, we further generated calibration curves, demonstrating that the prediction of OS and RFS by our nomograms was in close agreement with the actual outcomes (Figure 3E,F).

**FIGURE 4:**
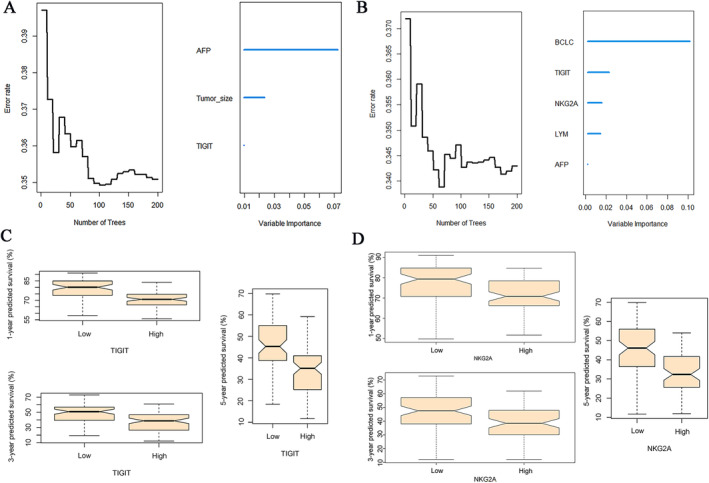
Examination for TIGIT and NKG2A in prognostic model. (A) Importance of variables in OS prognostic model. (B) Importance of variables in RFS prognostic model. (C) Predicted RFS with TIGIT expression. (D) Predicted RFS with NKG2A expression.

Moreover, employing the aforementioned model, we generated time‐dependent ROC curves of 1‐, 3‐, and 5‐ year OS and RFS, calculated the corresponding AUC, and conducted internal validation using bootstrapping (Figure 3G,H). The results revealed AUC values above 0.7 for each, suggesting the realism of our prognostic model. Given the strong predictive performance of our model, we further assessed the relative importance of each independent risk factor within the model using the random forest algorithm (Figure 4A,B). Although TIGIT expression demonstrated relatively lower importance in the OS prediction model, both TIGIT and NKG2A exhibited significant importance in RFS. Finally, we predicted the survival rates of patients at 1, 3, and 5 years based on the expression levels of NKG2A and TIGIT, respectively. Our findings indicated that patients with high NKGA2 and TIGIT expression had lower estimated survival rates at each time point compared to those with lower expression levels (Figure 4C,D), underscoring the negative prognostic impact of NKG2A and TIGIT expression.

## 4 Discussion

In our current investigation, we observed no correlation between TIGIT/NKG2A expression and clinical characteristics among HCC patients after liver resection. We identified highly expressed TIGIT as an independent risk factor impacting postoperative RFS and OS, whereas highly expressed NKG2A solely influenced postoperative RFS. In addition, we devised a prediction model founded on independent risk factors derived from multivariate analysis, thereby furnishing a theoretical framework for prognostic predictions among HCC patients following surgical intervention.

As a member of the poliovirus receptor protein family [14], TIGIT is expressed on human T cells and NK cells, functioning as an immune checkpoint that inhibits their activation [15, 16, 17, 18, 19]. Similar to the PD‐1/PD‐L1 axis, TIGIT exerts an immunosuppressive effect in tumor immune surveillance, thereby promoting the progression of HCC [20, 21]. Our study findings revealed that TIGIT expression in patients with HCC did not significantly correlate with tumor differentiation degree. However, previous research by Duan et al. [22] demonstrated a gradual increase in TIGIT expression with poorer HCC cell differentiation. This discrepancy may arise from variations in evaluation standards and sample size for immunohistochemistry results. In our study, we utilized the IRS score to assess immunohistochemistry results, stratifying them into low and high expression groups based on the score and employing a larger sample size. Consistent with prior investigations on TIGIT expression and cancer prognosis [23, 24, 25, 26], our Kaplan–Meier curve analysis demonstrated that patients with high TIGIT expression had shorter RFS and OS compared to those with low TIGIT expression. Furthermore, Cox multivariate analysis also confirmed that TIGIT expression level served as independent risk factors for both OS and RFS in patients after HCC surgery.

NKG2A, characterized by an intracytoplasmic tyrosine‐based inhibitory motif (ITIM), is also expressed on NK cells and T cells [27]. The interaction between NKG2A and human leukocyte antigen‐E (HLA‐E) serves to inhibit the activation of NK cells and T cells, and interrupting this interaction has been shown to enhance tumor immune response [27, 28]. In our current study, we did not observe a significant correlation between NKG2A expression and patients' clinical characteristics. However, Kaplan–Meier curve revealed that patients with low NKG2A expression exhibited better postoperative median RFS and OS compared to those with high expression levels. Additionally, Cox multivariate analysis confirmed that NKG2A expression level constituted an independent risk factor for RFS. This finding is consistent with the study by Sun et al. [29], which demonstrated that HCC patients with higher NKG2A expression exhibited features of NK cell depletion and experienced poorer prognosis, aligning with our results. Nevertheless, unlike TIGIT, NKG2A expression did not exhibit a significant correlation with OS in the multivariate analysis. This result may be attributed to sample size limitations, confounding factors, or the potential inclusion of deaths unrelated to HCC. Further research and discussion are warranted to elucidate these findings.

HCC presents a complex multifaceted pathology. While existing staging systems like BCLC stage and Child‐Pugh grade offer valuable insights for treatment planning and prognosis, achieving individualized assessment remains challenging [1, 2]. Advancements in cancer research and data analytics have led to the widespread adoption of tools such as the concordance index and nomograms, which amalgamate various clinical parameters to evaluate cancer prognosis. The concordance index adeptly gauges the prognostic model's alignment with real‐world outcomes, while nomograms translate intricate statistical data into user‐friendly graphical representations, enhancing the interpretability of predictive results [30, 31]. In our study, we identified independent prognostic factors for HCC patients through rigorous univariate and multivariate analyses. Leveraging these factors, we constructed a prognostic model for predicting the recurrence rate and mortality of 1, 3, and 5 years post‐operation. Evaluation of the model's performance via concordance index demonstrated consistency with actual outcomes, with values consistently exceeding 0.7 over the 1 to 5‐year period, affirming its predictive accuracy for OS and RFS. Visual representation of this model was achieved through nomograms, facilitating intuitive predictions of OS and RFS at different time points. Calibration curves further confirmed the reliability of our nomograms by demonstrating close alignment between predicted and actual values. Subsequent ROC curve analysis revealed AUCs exceeding 0.7 for 1‐, 3‐, and 5‐year OS and RFS predictions, underscoring the model's robustness, sensitivity, and specificity, with significant clinical implications. Furthermore, leveraging the random forest algorithm, we assessed the importance of NKG2A and TIGIT expression within the prognostic model. While TIGIT expression showed minimal impact on the OS prognostic model, both TIGIT and NKG2A emerged as crucial factors influencing RFS. Finally, we predicted the survival rates of patients at 1, 3, and 5 years based on the expression levels of TIGIT and NKG2A, respectively. Our findings indicated that patients with high TIGIT and NKG2A expression had lower estimated survival rates at each time point compared to those with lower expression levels, underscoring the negative prognostic impact of TIGIT and NKG2A expression.

Based on our prognostic model predicting recurrence and survival, patients exhibiting low expression levels of TIGIT and NKG2A demonstrated favorable prognoses, indicating a negative correlation between their expression and HCC prognosis. The development of prognostic models through multifactorial research enables individualized and precise prediction of HCC patient survival post‐surgery, representing a key trend in future clinical practice. Notably, TIGIT and NKG2A likely play pivotal roles in the onset and progression of HCC, suggesting their potential as immune targets for HCC treatment [32]. Several clinical trials targeting TIGIT, such as NCT06349980, NCT05775159, and NCT05757492, are currently underway to treat advanced or metastatic HCC patients.

However, our study has several limitations. Firstly, being a retrospective study, there may be bias in the process of case selection. Secondly, TIGIT and NKG2A are surface markers expressed on T cells and NK cells. Our immunohistochemical analysis only reflects the overall expression of these markers in tumor‐infiltrating T cells and NK cells, without differentiating their specific expression patterns within these cell types. Further investigation is warranted to explore this aspect. Thirdly, while our study confirmed the association between TIGIT and NKG2A expression and HCC prognosis, multivariate analysis only revealed a significant relationship between TIGIT expression and OS (*p* < 0.05), while NKG2A did not reach statistical significance (*p* = 0.073). Moreover, TIGIT expression did not play a crucial role in the established OS prognostic model. Lastly, although we have developed a relatively credible prognostic model, a larger sample size is needed to further validate its accuracy. It is imperative to expand the sample size, minimize biases, and extend the observation period to comprehensively analyze the relationship between TIGIT, NKG2A, and the prognosis of patients with HCC.

In conclusion, our study highlights the significance of both TIGIT and NKG2A expression as independent risk factors influencing postoperative RFS. While TIGIT emerges as an independent risk factor for postoperative OS, NKG2A does not. Moreover, we have successfully developed prognostic models for RFS, integrating pertinent independent risk factors such as TIGIT and NKG2A expression. These models offer enhanced predictive capability for the RFS outcomes of patients undergoing surgery for HCC.

## Author Contributions

**Junqi Wang:** conceptualization (equal), data curation (equal), investigation (equal), writing – original draft (equal), writing – review and editing (equal). **Yuqing Cao:** data curation (equal), formal analysis (equal), investigation (equal), methodology (equal), validation (equal), visualization (equal), writing – original draft (equal), writing – review and editing (equal). **Yu Tian:** funding acquisition (equal), project administration (equal), resources (equal), supervision (equal), writing – review and editing (equal). **Chaoliu Dai:** funding acquisition (equal), methodology (equal), project administration (equal), resources (equal), supervision (equal), writing – review and editing (equal). **Tianqiang Jin:** funding acquisition (equal),methodology (equal), resources (equal), writing – review and editing (equal). **Feng Xu:** conceptualization (equal), funding acquisition (equal), project administration (equal), resources (equal), supervision (equal), writing – review and editing (equal).

## Ethics Statement

The study protocol adhered to the ethical guidelines of the Helsinki Declaration and was approved by the Human Ethics Committee of Shengjing Hospital of China Medical University (approval number: 2021PS531K). Due to the retrospective design of the study, no written informed consent from patients was required, as decided by the local ethics committee. The application for exemption from informed consent has been approved.

## Conflicts of Interest

The authors declare no conflicts of interest.

## Supporting information


Data S1.

## Data Availability

The data that support the findings of this study are available from the corresponding author upon reasonable request.
