# Supplementary material for: A Novel Prognostic Nomogram Based on TIGIT and NKG2A Can Predict Relapse‐Free Survival of Hepatocellular Carcinoma After Hepatectomy
Source: Cancer Med. 2024 Nov 14;13(22):e70419. doi: 10.1002/cam4.70419 (PMC11561519; doi:10.1002/cam4.70419)
Supplement: Supplementary file 1 — Data S1. [file CAM4-13-e70419-s001.docx]

Supplementary Table 1. **correlation between TIGIT and NKG2A expression level**

|  | NKG2A^-^ | NKG2A^+^ | *P* |
| --- | --- | --- | --- |
| TIGIT ^-^ | 31 | 35 | 0.242 |
| TIGIT ^+^ | 45 | 33 |  |

Supplementary Table 2. **correlation between TIGIT, NKG2A expression and clinical characteristics**

| Clinical characteristics | TIGIT ^-^ | TIGIT ^+^ | *P value* | | | NKG2A ^-^ | | NKG2A ^+^ | *P value* | | |
| --- | --- | --- | --- | --- | --- | --- | --- | --- | --- | --- | --- |
|  | n=66 | n=78 | |  | | | n=76 | n=68 | | |  |
| Gender (female/ male) | 20(48.8%)/46(44.7%) | 21(51.2%)/57(55.3%) | | | 0.713 | 22(53.7%)/54(52.4%) | | 19(46.3%)/49(47.6%) | | 1.000 | |
| Age (<60/≥60y) | 41(50.0%)/25(40.3%) | 41(50.0%)/37(59.7%) | | | 0.311 | 44(53.7%)/32(51.6%) | | 38(46.3%)/30(48.4%) | | 0.867 | |
| BMI (<24/≥24) | 33(42.9%)/33(49.3%) | 44(57.1%)/34(50.7%) | | | 0.504 | 42(54.5%)/34(50.7%) | | 35(45.5%)/33(49.3%) | | 0.738 | |
| ASA grade |  |  | | |  |  | |  | |  | |
| Ⅰ | 4(33.3%) | 8(66.7%) | | |  | 4(33.3%) | | 8(66.7%) | |  | |
| Ⅱ | 51(47.2%) | 57(52.8%) | | |  | 62(57.4%) | | 46(42.6%) | |  | |
| Ⅲ | 11(45.8%) | 13(54.2%) | | | 0.708 | 10(41.7%) | | 14(58.3%) | | 0.862 | |
| Tumor number (single/ multiple) | 57(47.1%)/9(39.1%) | 64(52.9%)/14(60.9%) | | | 0.504 | 66(54.5%)/10(43.5%) | | 55(45.5%)/13(56.5%) | | 0.368 | |
| Tumor size(≤5/>5cm) | 44(45.4%)/22(46.8%) | 53(54.6%)/25(46.8%) | | | 1.000 | 55(56.7%)/21(44.7%) | | 42(43.3%)/26(55.3%) | | 0.214 | |
| Vascular cancer embolus (absent/present) | 61(46.2%)/5(41.7%) | 71(53.8%)/7(58.3%) | | | 1.000 | 70(53.0%)/6(50.0%) | | 62(47.0%)/6(50.0%) | | 1.000 | |
| Differentiation |  |  | | |  |  | |  | |  | |
| Poor | 2(33.3%) | 4(66.7%) | | |  | 2(33.3%) | | 4(66.7%) | |  | |
| Moderate-poor | 7(53.8%) | 6(46.2%) | | |  | 7(53.8%) | | 6(46.2%) | |  | |
| Moderate | 35(42.2%) | 48(57.8%) | | |  | 46(55.4%) | | 37(44.6%) | |  | |
| Moderate-high | 7(53.8%) | 6(46.2%) | | |  | 6(46.2%) | | 7(53.8%) | |  | |
| Well | 15(51.7%) | 14(48.3%) | | | 0.478 | 15(51.7%) | | 14(48.3%) | | 0.993 | |
| Liver Cirrhosis (absent/ present) | 4(28.6%)/62(47.7%) | 10(71.4%)/68(52.3%) | | | 0.259 | 7(50.0%)/69(53.1%) | | 7(50.0%)/61(46.9%) | | 1.000 | |
| Types of hepatitis |  |  | | |  |  | |  | |  | |
| Absent | 3(37.5%) | 5(62.5%) | | |  | 6(75.0%) | | 2(25.0%) | |  | |
| HBV | 54(44.6%) | 67(55.4%) | | |  | 62(51.2%) | | 59(48.8%) | |  | |
| Clinical characteristics | TIGIT ^-^ | TIGIT ^+^ | *P value* | | | NKG2A ^-^ | | NKG2A ^+^ | *P value* | | |
|  | n=66 | n=78 | |  | | | n=76 | n=68 | | |  |
| Steatohepatitis | 3(100.0%) | 0(0.0%) | | | 0.424 | 0(0.0%) | | 3(100.0%) | | 0.177 | |
| Hypersplenism (absent/ present) | 41(42.3%)/25(53.2%) | 56(57.7%)/22(46.8%) | | | 0.285 | 49(50.5%)/27(57.4%) | | 48(49.5%)/20(42.6%) | | 0.479 | |
| AFP level (<400/≥400ng/ml) | 49(47.1%)/17(42.5%) | 55(52.9%)/23(57.5%) | | | 0.710 | 57(54.8%)/19(47.5%) | | 47(45.2%)/21(52.5%) | | 0.461 | |
| Absolute value of lymphocyte  (<1.74/≥1.7×10^9/L) |  |  | | |  |  | |  | |  | |
| AFP level (<400/≥400ng/ml) | 39(42.4%)/27(51.9%) | 53(57.6%)/25(48.1%) | | | 0.299 | 48(52.2%)/28(53.8%) | | 44(47.8%)/24(46.2%) | | 0.864 | |
| Child-Pugh grade(A/B)  BCLC stage | 59(44.4%)/7(63.6%) | 74(55.6%)/4(36.4%) | | | 0.346 | 73(54.9%)/3(27.3%) | | 60(45.1%)/8(72.7%) | | 0.115 | |
|  |  |  | | |  |  | |  | |  | |
| 0 | 6(40.0%) | 9(60.0%) | | |  | 7(46.7%) | | 8(53.3%) | |  | |
| A | 49(47.1%) | 55(52.9%) | | |  | 58(55.8%) | | 46(44.2%) | |  | |
| B | 6(46.2%) | 7(53.8%) | | |  | 5(38.5%) | | 8(61.5%) | |  | |
| C | 5(41.7%) | 7(58.3%) | | | 0.934 | 6(50.0%) | | 6(50.0%) | | 0.705 | |
|  |  |  | | |  |  | |  | |  | |
|  |  |  | | |  |  | |  | |  | |
